# Supplementary material for: Neuromuscular screening and cognitive function in older adults: A cross-sectional exploratory study
Source: PLoS One. 2026 Feb 20;21(2):e0343404. doi: 10.1371/journal.pone.0343404 (PMC12923004; doi:10.1371/journal.pone.0343404)
Supplement: Attachment — Submitted filename: comments Review.docx [file pone.0343404.s004.docx]

Neuromuscular Screening and Cognitive Function in Older Adults is an interesting exploratory cross‑sectional study but it has important limitations in design, reporting, and interpretation that need addressing from authors before it fits journal.

1. It has a very small, convenience sample (n=20), all from a university lifelong-learning course, severely limits external validity and makes claims about “early detection” or “screening” premature and overstated.

2. Sample size justification is a concern, citing “similar pilot investigations” without references or target effect size is weak.

3. Eligibility criteria are minimal with no screening for neurological, psychiatric, major cardiovascular, or neuromuscular conditions that can directly affect both cognition and muscle function

4.Methods : there is no detail on electrode placement landmarks, pulse train structure, exact torque sensor specs, or calibration/verification of the custom device. Is the device checked for Reliability and Validity? Add a substantially more detailed Methods subsection for the neuromuscular device and stimulation protocol, sufficient to reproduce the setup.

5. Results : The statement that fat mass and percent body fat are “elevated compared to normative values” is not backed by explicit normative references or statistical comparisons and therefore sounds speculative.

Reported “mean total MET‑min/week: 125.4 ± 82.2 (range 6.6–334.6)” is orders of magnitude lower than standard IPAQ‑SF MET‑min/week values (which typically run in the thousands), suggesting either that units are not standard or that a transformation has been applied.

Please clarify:

Exactly how IPAQ‑SF data were processed and whether the standard MET‑constants and summation procedures were followed.

The units and scaling used in Table 1 (are these total MET‑hours, averages per day, or rescaled values?).

6. Please: Provide explicit numbers on those invited, declined, and excluded (if available), consistent with STROBE for observational studies.
