# Supplementary material for: Neuromuscular screening and cognitive function in older adults: A cross-sectional exploratory study
Source: PLoS One. 2026 Feb 20;21(2):e0343404. doi: 10.1371/journal.pone.0343404 (PMC12923004; doi:10.1371/journal.pone.0343404)
Supplement: Attachment — Submitted filename: Response to Reviewers.docx [file pone.0343404.s005.docx]

Dear Academic Editor and Reviewers,

We would like to thank you for your careful evaluation of our manuscript and for the constructive and insightful comments provided. We appreciate the time and effort invested by the reviewers, which helped us to improve the clarity, methodological transparency, and interpretation of our study.

We have revised the manuscript accordingly and have addressed each comment point by point below. All changes made to the manuscript are highlighted in the revised version with track changes.

___________________________________________________________________________

**Reviewer #1:** **Neuromuscular Screening and Cognitive Function in Older Adults is an interesting exploratory cross‑sectional study but it has important limitations in design, reporting, and interpretation that need addressing from authors before it fits journal.**

**1. It has a very small, convenience sample (n=20), all from a university lifelong-learning course, severely limits external validity and makes claims about “early detection” or “screening” premature and overstated.**

The manuscript has been revised to more explicitly frame the study as exploratory and hypothesis-generating. References to screening have been replaced with more cautious terminology (e.g., assessment framework, feasibility, proof-of-concept), and the limitations regarding external validity have been strengthened accordingly.

**2. Sample size justification is a concern, citing “similar pilot investigations” without references or target effect size is weak.**

We thank the reviewer for this comment. A reference to similar pilot investigations has now been added to the manuscript to support the sample size justification.

**3. Eligibility criteria are minimal with no screening for neurological, psychiatric, major cardiovascular, or neuromuscular conditions that can directly affect both cognition and muscle function**

The Methods section has been revised to explicitly state that no formal clinical screening for neurological, psychiatric, cardiovascular, or neuromuscular conditions was performed.

**4. Methods: there is no detail on electrode placement landmarks, pulse train structure, exact torque sensor specs, or calibration/verification of the custom device. Is the device checked for Reliability and Validity? Add a substantially more detailed Methods subsection for the neuromuscular device and stimulation protocol, sufficient to reproduce the setup.**

Specifically, we added a detailed description of bipolar electrode placement over the motor point of the tibialis anterior muscle (and the pretibial muscle group). We further expanded the description of the stimulation waveform and parameters (biphasic pulses with a duration of 200 µs, stimulation intensity starting at 30 mA and increased by 5 mA according to individual tolerance using a constant-current stimulator, stimulation duration of 4 s), as well as the procedure for torque measurement and force calculation. In addition, information on device calibration and validation has been included in the Methods section.

**5. Results: The statement that fat mass and percent body fat are “elevated compared to normative values” is not backed by explicit normative references or statistical comparisons and therefore sounds speculative.** We agree with the reviewer that comparative statements with normative values are not appropriate in the Results section. The phrase “compared to age- and sex-matched normative values” has therefore been removed, and the Results are now presented in a purely descriptive manner without external comparisons.

Reported “mean total MET‑min/week: 125.4 ± 82.2 (range 6.6–334.6)” is orders of magnitude lower than standard IPAQ‑SF MET‑min/week values (which typically run in the thousands), suggesting either that units are not standard or that a transformation has been applied.

**Please clarify:**

**Exactly how IPAQ‑SF data were processed and whether the standard MET‑constants and summation procedures were followed.**

**The units and scaling used in Table 1 (are these total MET‑hours, averages per day, or rescaled values?).**

We thank the reviewer for identifying this issue. We acknowledge that the originally reported IPAQ-SF values were derived using a non-standard unit conversion, which resulted in values that were not comparable to standard MET-min/week reporting. This has been corrected. Importantly, this correction did not affect the reported correlations.

**6. Please: Provide explicit numbers on those invited, declined, and excluded (if available), consistent with STROBE for observational studies.**

The Methods section has been revised. Participants were recruited using a convenience sampling approach within a lifelong-learning program; a larger group was informed about the study, and the first individuals who expressed interest and met the inclusion criteria were enrolled. All individuals who registered for participation completed the full assessment protocol, with no dropouts.

___________________________________________________________________________

**Reviewer #2:** This is a highly intriguing prospective study that establishes an association between two critical factors influencing disordered aging in older people—cognitive impairment and muscle function decline—while incorporating body composition as a control. However, potential issues with sample size and experimental design reliability resulted in largely non-significant findings. The study merits publication.

Suggestions for revision:

**1. Describe the basic characteristics of the subjects, including inclusion criteria, age range, and sociodemographic attributes of the population;**

The Methods section has been revised to provide a clearer description of participant characteristics, including inclusion criteria, age range, sex distribution, and recruitment context.

**2. Justify the selection of 16 Hz and 60 Hz electrical stimulation frequencies.**

We thank the reviewer for this comment. The rationale for selecting stimulation frequencies of 16 Hz and 60 Hz has now been explicitly added to the Methods section (Neuromuscular function assessment). Briefly, the two frequencies were chosen to capture different aspects of neuromuscular activation, with the lower frequency representing a low-frequency neuromuscular response and the higher frequency eliciting a more pronounced high-frequency contractile response, consistent with the exploratory nature of the study.

**3. Ensure appropriate methods for correlating continuous and discrete variables;**

The Data Analysis section has been revised to explicitly describe the correlation methods used for continuous variables and to clarify that discrete variables were not included in correlational analyses.

**4. Due to excessively high standard deviation values, data reliability analysis is required, and unreliable data should be excluded.**

We would like to clarify that the relatively large standard deviations observed in several variables reflect substantial inter-individual variability within this heterogeneous older adult sample rather than measurement unreliability. No test–retest reliability analysis was planned, as the study was not designed as a reliability study and all measurements were obtained using standardized procedures and calibrated instruments. No data points met predefined criteria for exclusion, and therefore no values were removed from the analyses. This clarification has been added to the Data Analysis section.

**5. While sample size requirements are less stringent for exploratory studies, statistical analysis remains necessary to ensure the reliability of the results.**

We agree with the reviewer that appropriate statistical analysis is essential even in exploratory studies. Accordingly, all analyses were conducted using established statistical methods suitable for small samples, including descriptive statistics, non-parametric correlation analyses, and exploratory regression models.

**6. Gender factors persist.**

We agree that sex-related factors may influence both neuromuscular and cognitive outcomes. This limitation has been explicitly acknowledged.

**Research Methodology Questions:**

**1. What is a “custom-built” device?**

The device is being developed by a certified manufacturer and has been tested at the research laboratory of the University Hospital Hradec Králové as part of a project approved by the Ethics Committee for the objective measurement of muscle strength in a healthy population. The safety of the device is guaranteed by a Declaration of Conformity.

**2. Why was the tibialis anterior muscle selected as the test site?**

The device can be used to test large muscle groups in both the upper and lower limbs. The tibialis anterior was chosen because it is an easily stimulable and anatomically accessible muscle, for which a normative database was established based on testing of 192 healthy subjects.

**3. What is the anatomical location for “targeting the motor point within the anterior compartment of the lower leg”?**

It is located one-third (1/3) of the way down a straight line drawn from the tibial tuberosity  to the medial malleolus, one electrode is placed over this motor point, while the second (reference) electrode is placed 3 cm distally along the same muscle fibers.
